# Evaluation of the quality of clinical guidelines for prophylaxis of venous thromboembolism in urological surgeries by the AGREE II review instrument

**DOI:** 10.1002/hsr2.1118

**Published:** 2023-02-16

**Authors:** Behnam Shakiba, Ali Kabir, Shirin Irani, Nasim Torabi, Vahid Nourmohamad, Mohaddese Farid

**Affiliations:** ^1^ Department of Urology, School of Medicine, Firoozgar Hospital Iran University of Medical Sciences Tehran Iran; ^2^ Firoozgar Clinical Research Development Center Iran University of Medical Sciences Tehran Iran; ^3^ Associate Professor of Epidemiology, Minimally Invasive Surgery Research Center Iran University of Medical Sciences Tehran Iran; ^4^ Otorhinolaryngology Research Center, Amiralam Hospital, School of medicine Tehran University of Medical Sciences Tehran Iran; ^5^ Department of Cardiology, School of Medicine, Firoozgar Hospital Iran University of Medical Sciences Tehran Iran; ^6^ School of Medicine Tabriz University of Medical Sciences Tabriz Iran

**Keywords:** AGREE II, clinical guidelines, urological surgery, venous thromboembolism

## Abstract

**Background and Aims:**

Venous thromboembolism (VTE) is the most common cause of death during the first 30 days after surgery. There is not any study which critically evaluated clinical guidelines related to VTE prophylaxis in urological surgeries. Therefore, in this study, we decided to evaluate related clinical guidelines using the AGREE II instrument to take a positive step towards improving the care of these patients.

**Methods:**

The latest version of all available clinical guidelines related to the topic of VTE prophylaxis in urological surgeries until 2021 was searched. Four appraisers, including one urologist, one cardiologist, one epidemiologist, and one MD who had prior knowledge of working with the AGREE II tool and international articles in this field appraised selected clinical guidelines. Using the AGREE II review tool, clinical guidelines were critically evaluated. Then, the score of six domains of AGREE II for each guideline was calculated and compared with each other, and the relationship between the domains was measured by Kendall's correlation test. To determine the reliability of the test, interclass correlation coefficients were calculated for all indicators.

**Results:**

Items were rated on a 7‐point scale from 1 (strongly disagree) to 7 (strongly agree). NICE, CHEST, and EAU guidelines obtained the highest scores from the Overall Assessment criteria by scoring 6, 5.75, and 5.25, respectively. There was only a correlation between the score of Overall Assessment criterion with “Applicability” domain, with Kendall's correlation coefficient of 0.867 and *p* = 0.015. The domains of “Clarity and presentation” and “Scope and purpose” obtained the highest standardized scores by getting 84.49% and 75.69%, respectively, and “Applicability” with 30.04% obtained the lowest standardized score.

**Conclusion:**

In this study, NICE, CHEST, and EAU guidelines are suggested as clinical guidelines by obtaining the highest scores from Overall Assessment criterion.

## 1 INTRODUCTION

The goal of evidence‐based medicine is to encourage medical doctors to make rational decisions based on the best and most up‐to‐date evidence. In the meantime, clinical practice guidelines (CPG) play the main role by providing clinical decision‐making choices, according to scientific evidence and current facilities.^1^ The available evidence is sometimes limited and sometimes contradictory, and on the other hand, the facilities of clinical centers are also different in different regions. Therefore, transferring research results to the clinical field and providing practical solutions is not an easy task.^2^ We need more than simple drug prescriptions to make decisions and judgments; Such as profit and loss, patient's wishes, economic problems, etc. Therefore, to create practical solutions, it is necessary to discuss and spend lots of energy between all the people related to that particular issue.^3^ In fact, CPGs are strategies that have been systematically developed and help doctors to make decisions about a specific issue.^4^

In recent years, the creation of various clinical guidelines for different topics has grown a lot.^5^ So that currently these CPGs exist in almost all aspects of clinical practice and health policy. In fact, the main goal of these clinical guidelines is to increase the practical use of the results obtained from the research.^6^ Due to the large number of clinical guides in different subjects, medical doctors and researchers who intend to use these clinical guides will be lost in choosing a good clinical guideline.^5^

On the other hand, venous thromboembolism (VTE), which consists of two components, deep vein thrombosis and pulmonary embolism, is a life‐threatening complication after surgeries and the most common cause of death during the first 30 days after surgeries.^7^ Considering that the risk of VTE occurrence can be reduced through medical and clinical prophylaxis,^8^ the way of management and the importance of using prophylaxis of VTE in urological surgeries are noticable.

Evidence that have examined VTE prophylaxis in urological surgeries are limited^9^ and so far no study has critically evaluated clinical guidelines related to VTE prophylaxis in urological surgeries. Therefore, in this study, we decided to examine and evaluate related clinical guidelines using the AGREE II instrument to take a positive step towards improving the care of these patients.

## 2 MATERIALS AND METHODS

### 2.1 Identification of the guidelines

First, the latest version of all available clinical guidelines related to the topic of VTE prophylaxis in urological surgeries until 2021 was searched. Medline (by PubMed), Google Scholar, Embassy and Trip database were used to search for clinical guidelines, and Expert Contact was also used for additional search. The entry criteria were the latest version of national and international guidelines on the topic of prophylaxis of VTE in urological surgeries until 2021, which had a valid version in English or French. The Persian guidelines were also searched through national databases, all Iranian urology and cardiology research centers' web sites and via personal contacts with experts.

We included guidelines with an exclusive or predominant focus on this topic and the decision to include or exclude a guideline was made by two authors independently. Any disagreements were resolved by consensus.

### 2.2 Appraisal instrument

The AGREE II instrument consists of 23 items categorized in six domains:

1. *Scope and purpose*: three items that address the overall aim of the guideline, the clinical question and the target population.
2. *Stakeholder involvement*: four items addressing the composition, expertize and representation of the development group.
3. *Rigor of development*: seven items that evaluate the process of locating and synthesizing the evidence, and formulating and updating the recommendations.
4. *Clarity and presentation*: four items that address language and format.
5. *Applicability*: three items that address the potential organizational, behavioral and cost implications of applying the guidelines.
6. *Editorial independence*: two items that focus on potential conflicts of interest.

Items are rated on a 7‐point scale from 1 (strongly disagree) to 7 (strongly agree). The final item of the instrument asks the appraiser to consider the overall quality of the clinical guideline and decide whether they recommend the guideline for practice.

### 2.3 Participant

Four appraisers, including one urologist, one cardiologist, one epidemiologist, and one MD who had prior knowledge of working with the AGREE II tool and international articles in this field appraised the clinical guidelines. They were all familiar with the fundamentals of evidence‐based medicine and clinical research methodology. All appraisers attended a workshop that covered the basic principles of developing valid clinical guidelines and a detailed presentation of the AGREE II instrument.

### 2.4 Quality assessment

Each clinical guideline was assessed by four appraisers; all of them used the English version of the AGREE II. The clinical guidelines were appraised and scored independently by the appraisers.

The Persian version of the AGREE II tool has been validated and shown to provide scores that are comparable with the original tool.^10^

### 2.5 Data analysis

Domain scores of each clinical guideline were standardized following the method recommended by the AGREE II^11^ and compared among the guidelines. The intraclass correlation coefficients (ICCs) were compared among different numbers of appraisers. The ICCs above 0.75 were considered to represent good, 0.40–0.75 moderate and below 0.40 poor reliability.^12^, ^13^ The Kendall's tau correlation analysis was done to assess the correlation between the domain scores and the overall assessment. Analysis of variance tests were used to compare the overall assessment scores among the guidelines. We hypothesized that the number of algorithms in the guidelines was an important determinant of the overall assessment score and used Kendall correlation tests to assess this hypothesis.

## 3 RESULTS

### 3.1 Characteristics of clinical guidelines

We included six clinical guidelines in our study (Box 1). No French or Persian guidelines were added and the publication year of the guidelines was from 2009 to 2021. In fact, Canadian Urological Association Guideline is an adaption of EAU guideline.

**Clinical guidelines for prophylaxis of venous thromboembolism in urological surgeries**

**Table:** 

Row	Abbreviation name	Publication source	Title clinical guide	Pub. Year
1	AUA	American Urological Association	Thromboprophylaxis guidelines for patients undergoing urological surgery	2009
2	CHEST	American College of Chest Physicians	Prevention of VTE in nonorthopedic surgical patients antithrombotic therapy and prevention of thrombosis, 9th ed	2012
3	CUA	Canadian Urological Association	Perioperative thromboprophylaxis and management of anticoagulation	2021
4	EAU	European Association of Urology	EAU Guidline on thromboprophylaxis in urological surgery	2017
5	NICE	The National Institute for Health and Care Excellence	Venous thromboembolism in over 16s: reducing the risk of hospital‐acquired deep vein thrombosis or pulmonary embolism	2018
6	SIGN	Scottish Intercollegiate Guidelines Network	Urology Surgery—reducing the risk of venous thromboembolism	2014

### 3.2 AGREE scores

The mean standardized scores of the guidelines' domains and items have been shown in Table 1. Items were rated on a 7‐point scale from 1 (strongly disagree) to 7 (strongly agree). “Clarity and presentation” and “Scope and purpose” achieved the highest scores among the AGREE domains with the mean standardized scores of 84.49% and 75.69%, respectively. “Applicability” got the lowest score with the mean of 30.04%. We observed statistically significant differences between the domain score of “Rigor of development” with “Scope and purpose” (*p* = 0.022), “Clarity and presentation” (*p* = 0.032) and “Applicability” (*p* = 0.039). There was also statistically significant difference between the domain score of “Stakeholder involvement” and “Applicability” (*p* = 0.039). No statistically significant difference among other domain scores was found.

**Table 1:** Quality assessment of the guidelines prophylaxis of venous thromboembolism in urological surgeries according to AGREE II instrument (values are presented as mean standard deviation [SD]).

Guidelines	AUA	CHEST	CUA	EAU	NICE	SIGN	Mean
Domain 1: Scope and purpose							
1. The overall objective(s) of the guideline is (are) specifically described	6.75	5.25	6.50	6.25	6.25	6.50	6.25
	(0.50)	(1.26)	(1.00)	(0.96)	(0.96)	(1.00)	(0.52)
2. The clinical question(s) covered by the guideline is (are) specifically described	4.57	7.00	3.25	3.25	7.00	5.50	5.13
	(1.26)	(0.00)	(1.50)	(2.06)	(0.00)	(1.29)	(1.69)
3. The patients to whom the guideline is meant to apply are specifically described	5.00	6.50	3.75	4.00	6.25	6.00	5.25
	(1.15)	(0.58)	(0.96)	(2.16)	(0.96)	(0.82)	(1.18)
Total standardized score (percentages)	75.00	87.50	58.33	58.33	91.67	83.33	75.69
Domain 2: Stakeholder involvement							
4. The guideline development group includes individuals from all the relevant professional groups	1.75	4.75	1.75	5.50	5.75	5.75	4.21
	(0.50)	(1.26)	(0.96)	(1.73)	(2.50)	(2.50)	(1.94)
5. The patients' views and preferences have been sought	1.00	3.50	2.25	1.75	4.00	5.75	3.04
	(0.00)	(2.65)	(2.50)	(0.96)	(2.00)	(2.50)	(1.73)
6. The target users of the guideline are clearly defined	2.00	3.50	2.25	5.50	6.00	5.75	4.17
	(1.41)	(2.38)	(0.96)	(2.38)	(0.82)	(2.50)	(1.81)
7. The guideline has been piloted among end users	2.25	6.50	1.00	3.25	5.75	4.50	3.88
	(1.50)	(0.58)	(0.00)	(2.87)	(1.50)	(2.38)	(2.10)
Total standardized score (percentages)	9.72	48.61	18.06	54.17	70.83	79.17	46.76
Domain 3: Rigor of development							
8. The systematic methods were used to search for evidence	1.75	4.50	1.25	4.25	4.75	3.00	3.25
	(0.96)	(1.00)	(0.50)	(2.75)	(0.50)	(1.41)	(1.49)
9. The criteria for selecting the evidence are clearly described	1.00	7.00	1.75	5.75	4.25	3.25	3.83
	(0.00)	(0.00)	(0.96)	(0.50)	(0.50)	(1.71)	(2.31)
10. The methods used for formulating the recommendations are clearly described	1.75	6.00	4.25	5.00	5.00	2.75	4.13
	(0.96)	(0.00)	(0.50)	(1.83)	(1.15)	(1.26)	(1.59)
11. The health benefits, side effects and risks have been considered in formulating the recommendations	5.50	6.50	4.00	6.25	6.25	5.25	5.63
	(1.73)	(0.58)	(2.16)	(0.96)	(0.96)	(2.87)	(0.93)
12. There is an explicit link between the recommendations and the supporting evidence	3.00	7.00	1.25	4.25	6.00	5.25	4.46
	(2.16)	(0.00)	(0.50)	(2.75)	(0.82)	(2.87)	(2.09)
13. The guideline has been externally reviewed by experts before its publication	2.25	1.00	1.00	1.00	4.50	5.50	2.54
	(0.96)	(0.00)	(0.00)	(0.00)	(2.89)	(3.00)	(1.99)
14. A procedure for updating the guideline is provided	1.00	1.00	1.25	1.00	4.00	5.75	2.33
	(0.00)	(0.00)	(0.50)	(0.00)	(2.94)	(2.50)	(2.05)
Total standardized score (percentages)	21.88	65.63	16.15	47.40	67.71	56.77	45.92
Domain 4: Clarity and presentation							
15. The recommendations are specific and unambiguous	5.25	6.75	4.50	6.25	6.75	5.50	5.92
	(2.06)	(0.50)	(0.00)	(0.96)	(0.50)	(2.38)	(0.63)
16. The different options for management of the condition are clearly presented	5.75	6.75	4.50	6.25	6.75	5.50	5.92
	(2.50)	(0.50)	(1.29)	(0.96)	(0.50)	(3.00)	(0.86)
17. Key recommendations are easily identifiable	5.50	7.00	5.75	6.50	7.00	5.50	6.21
	(2.38)	(0.00)	(0.50)	(1.00)	(0.00)	(3.00)	(0.71)
18. The guideline is supported with tools for application	1.25	3.75	1.50	1.50	4.25	2.75	2.50
	(0.50)	(0.96)	(1.00)	(1.00)	(1.71)	(2.06)	(1.28)
Total standardized score (percentages)	75.00	97.22	73.61	88.89	97.22	75.00	84.49
Domain 5: Applicability							
19. The potential organizational barriers in applying the recommendations have been discussed	3.75	2.25	1.25	4.25	6.00	5.00	3.75
	(1.50)	(1.89)	(0.50)	(3.20)	(1.41)	(2.45)	(1.75)
20. The potential cost implications of applying the recommendations have been considered	1.00	1.75	1.00	2.00	5.00	3.00	2.29
	(0.00)	(0.96)	(0.00)	(1.41)	(1.83)	(2.31)	(1.52)
21. The guideline presents key review criteria for monitoring and/or audit purposes	1.00	1.75	1.00	2.00	4.75	5.50	2.67
	(0.00)	(0.96)	(0.00)	(1.41)	(1.71)	(3.00)	(1.96)
Total standardized score (percentages)	12.50	22.92	3.13	23.96	66.67	51.04	30.04
Domain 6: Editorial independence							
22. The guideline is editorially independent from the funding body	4.00	6.75	3.50	6.25	2.25	4.25	4.50
	(2.16)	(0.50)	(1.29)	(0.96)	(2.50)	(2.22)	(1.70)
23. Conflicts of interest of guideline development members have been recorded	5.75	6.75	6.25	7.00	1.75	5.00	5.42
	(1.26)	(0.50)	(0.50)	(0.00)	(1.50)	(2.71)	(1.93)
Total standardized score (percentages)	64.58	95.83	64.58	93.75	16.67	60.42	65.97
Overall assessment	3.75	5.75	3.25	5.25	6.00	4.75	4.79

**Figure 1:**
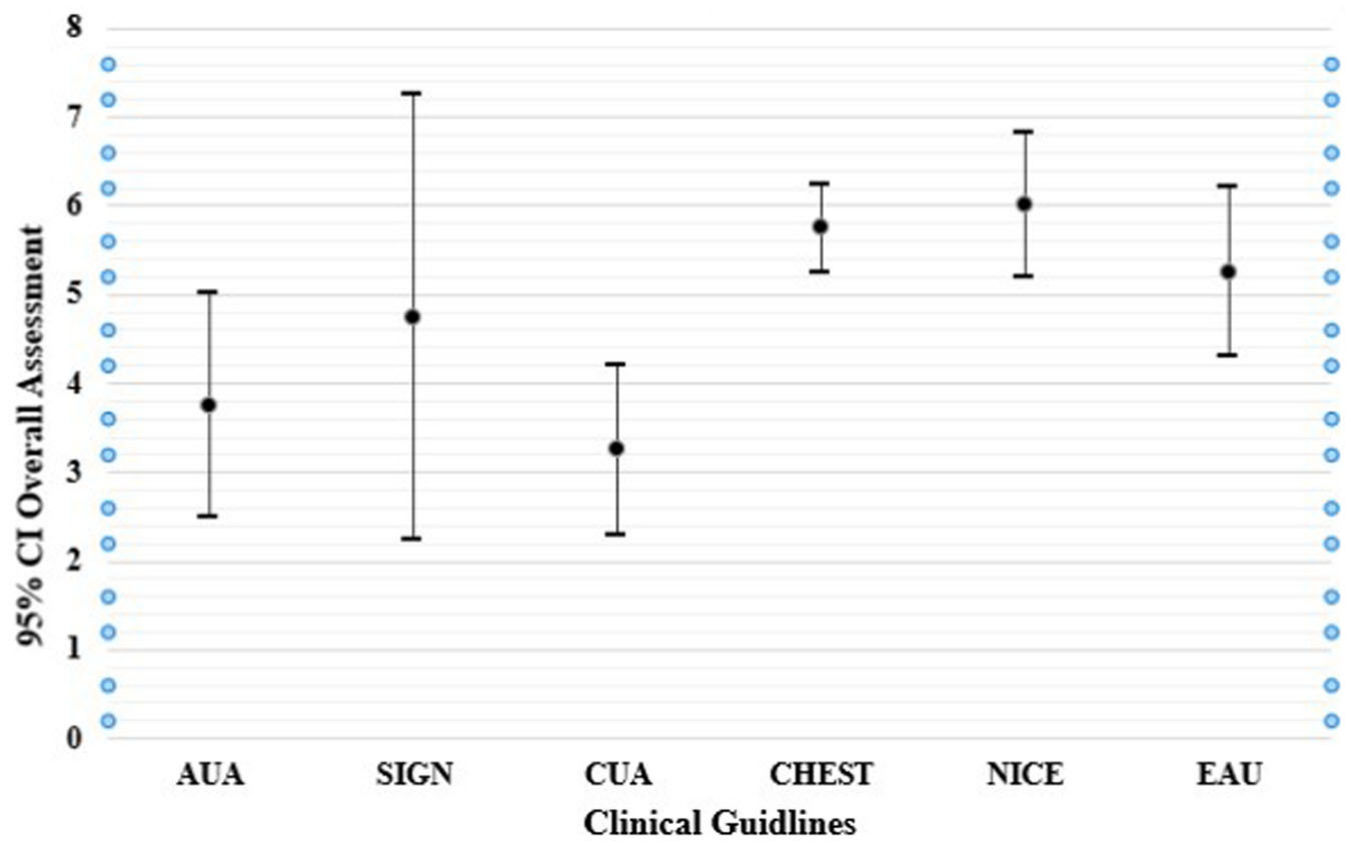
The overall assessment source among included guidelines.

The overall assessment scores were compared between clinical guidelines and NICE, CHEST, and EAU obtained the highest scores (Figure 1). Table 2 shows the correlations between the overall assessment and the domain scores. Only domain of “Applicability” (*p* = 0.015) had a statistically significant correlation with the overall assessment score (Kendall's tau = 0.867).

**Table 2:** Intraclass correlation coefficients for each AGREE instrument domain as a function of the number of raters and correlation coefficients between overall assessment scores and each AGREE domain score.

Domains	Scope and purpose	Stakeholder involvement	Rigor of development	Clarity and presentation	Applicability	Editorial independence
Intraclass correlation coefficients						
1 Rater	0.055	0.454	0.522	0.240	0.563	0.442
2 Raters	0.653	0.579	0.623	0.504	0.668	0.452
3 Raters	0.769	0.696	0.744	0.509	0.778	0.656
4 Raters	0.830	0.769	0.814	0.558	0.837	0.768
Correlation coefficients with overall assessment (Kendall's tau)						
Overall assessment	0.251	0.091	0.091	0.079	0.015*	0.444

* Correlation is significant at *p* < 0.05.

**Figure 2:**
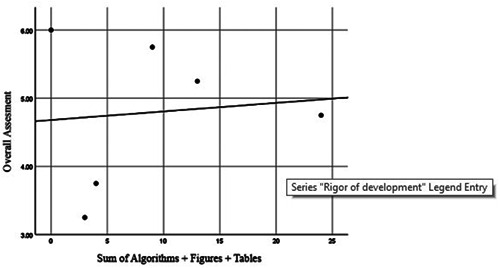
Correlation between the overall assessment scores and the total number of algorithms, tables, and figures in the guideline.

We proposed the hypothesis that the “easy identification of key recommendations and application tools” may play a role in the appraisers' perspective towards a guideline but there was no significant relationship between the overall assessment scores and the total numbers of algorithms, tables, and figures in guidelines (Kendall's tau = −0.067, *p* = 0.851) (Figure 2).

### 3.3 AGREE validity and reliability assessment

**Figure 3:**
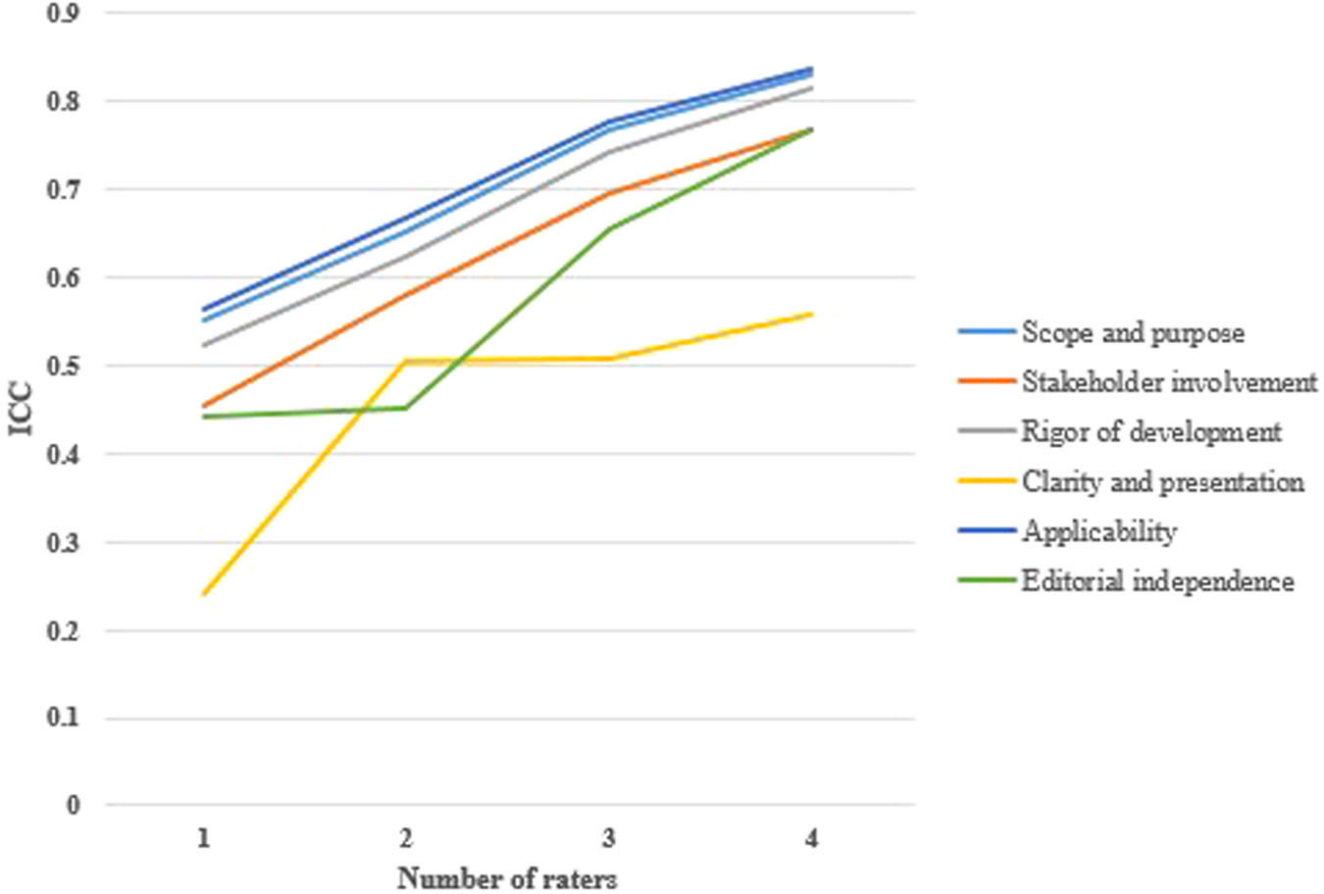
Intraclass correlation coefficients for each AGREE instrument domain as a function of the number of raters.

The ICCs for each AGREE domain are shown in Table 2. “Applicability” had the highest reliability score (0.837), followed by “Scope and purpose” (0.830) and “Rigor of development” (0.814). Table 2 demonstrates that no domain's ICC reached 0.75, if a single or two appraisers performed the evaluation. If three appraisers were involved, in two domains (“Applicability” and “Scope and purpose”) ICC reached 0.75 but increasing the number of appraisers from three to four did not improve the ICC values of “Clarity and presentation” to the predefined value of 0.75 (Figure 3).

## 4 DISCUSSION

Various studies have applied the AGREE instrument for assessing the quality of guidelines for a specific subject. To our knowledge, this is the first systematic evaluation of the quality of clinical guidelines for the management of prophylaxis of VTE in urological surgeries.

In our study NICE, CHEST, and EAU clinical guidelines obtained higher overall assessment scores in the appraisal process and were recommended for use in practice. Among these three clinical guidelines, the NICE guideline obtained the highest standardized score in three domains of “Scope and purpose,” “Rigor of development,” and “Applicability” and the CHEST guideline obtained the highest standardized score in domain of “Editorial independence.” Also the CHEST and the NICE guidelines obtained the highest standardized score in domain of “Clarity and presentation” together. But the EAU guideline, despite the third ranking in term of Overall assessment score, did not achieve the highest standardized score in any of six domains.

“Applicability” was the only domain with a statistically significant correlation with the overall assessment score (Kendall's tau = 0.867, *p* = 0.015). While in Irani et al.'s^14^ and MacDermid et al.'s^12^ results, the strongest correlations of Overall Assesment was with domain of “Clarity and presentation.” It shows that the potential organizational, behavioral and cost implications of applying the guidelines play an important role in their quality and overall value in our study.

The domains of “Clarity and presentation” and “Scope and purpose” obtained the highest standardized scores by getting 84.49% and 75.69%, respectively in our study. These two domains obtained the highest standardized score in a study conducted by Irani et al. in 2010^14^ on evaluating the CPGs developed for the management of thyroid nodules and thyroid cancers using the AGREE review instrument^14^ and also in a study conducted by Cates et al. on occupational medicine clinical guidelines.^15^

“Scope and purpose” is a domain of AGREE II instrument, which has been assigned the highest standardized score in most studies; For example, in the study of Hulshof and Hoenen in 2007, this domain obtained the highest standardized score (87%) in review of guidelines used by occupational medicine specialists^16^ and also in the study of Appleyard et al. in 2006, this domain obtained the highest standardized score (68%) in review of clinical guidelines for pelvic pain related to endometriosis.^17^

In contrast, the “cost implications” and the “criteria for monitoring and/or audit purposes” (applicability domain) had the lowest standardized score (30.04%) among the 6 domains examined in our study. Not giving attention to the applicability issues is a chronic problem that exists in many guidelines.^18^ In the study of Hulshof and Hoenen in 2007, this domain obtained the lowest score with 53%^16^ and also in the study of Appleyard et al. in 2006, “Applicability” with a percentage of only 14%, had the lowest standardized score.^17^ This low score of “Applicability” domain, along with the significant relationship of this domain with the criterion of Overall Assesment in this study can indicate the biggest weakness of the guidelines reviewed by appraisers. This result suggests the writers of guidelines to focus more on the evaluation of organizational and behavioral aspects and the possible costs of using clinical guidelines in real clinical use.

AGREE recommends a minimum of two and preferably four raters to evaluate clinical guidelines with this instrument.^11^ The results of our study do not confirm the existence of two appraisers in context of any domain and if the opinions of three raters are used, only in two domains of “Applicability” and “Scope and purpose,” ICC would be in the reliability range. Therefore, our study considers the presence of at least 4 raters for evaluation of clinical guidelines by the AGREE II review instrument. While in the study of Irani et al. in 2010, this number was estimated to be at least 3.^14^

In our study, no significant statistical relationship was found between the total number of algorithms, images and tables in the guidelines, with the criterion score of Overall Assesment (Kendall's correlation coefficient = −0.067 with *p* = 0.851). For example, the NICE guideline did not use any algorithms, images or tables in presenting its clinical guidelines, despite obtaining the highest score from the Overall Assesment criterion. Therefore, in this study and according to four appraisers, the overall value and validity of each guideline was independent from the number of algorithms, images and tables.

## 5 CONCLUSION

There was only a correlation between the score of Overall Assesment criterion with “Applicability” domain, with Kendall's correlation coefficient of 0.867 and *p* = 0.015, and this result shows the importance of the “Applicability” domain in evaluating clinical guidlines. The low percentage of the “Applicability” domain, along with the significant relationship of this domain with the Overall Assesment criterion, can indicate the greatest weakness of the guidelines reviewed in this study, and it suggests the writers of guidelines to focus more on the evaluation of organizational and behavioral aspects and the possible costs of using clinical guidelines in real clinical use.

In our study NICE, CHEST, and EAU clinical guidelines obtained higher overall assessment scores in the appraisal process and were recommended for use in practice. Although, the EAU guideline has the third ranking in term of Overall assessment score, but this guideline is urology procedure‐specific. In considering these factors, we felt it would be appropriate to use by urologists.

## AUTHOR CONTRIBUTIONS

**Behnam Shakiba**: Conceptualization; data curation; supervision; writing—review and editing. **Ali Kabir**: Conceptualization; data curation; writing—review and editing. **Shirin Irani**: Data curation; writing—review and editing. nasim torabi: conceptualization; data curation; supervision; writing—review and editing. **Vahid Nourmohamad**: Methodology; writing—original draft. **Mohaddese Farid**: Methodology; writing—original draft.

## CONFLICTS OF INTEREST STATEMENT

Behnam Shakiba is an Editorial Board member of Health Science Reports and coauthor of this article. He was excluded from editorial decision‐making related to the acceptance of this article for publication in the journal. The remaining authors declare no conflict of interest.

## TRANSPARENCY STATEMENT

The lead author Nasim Torabi affirms that this manuscript is an honest, accurate, and transparent account of the study being reported; that no important aspects of the study have been omitted; and that any discrepancies from the study as planned (and, if relevant, registered) have been explained.

## Data Availability

The authors confirm that the data supporting the findings of this study are available within the article.
